# Molecular and Pathological Features of Gastric Cancer in Lynch Syndrome and Familial Adenomatous Polyposis

**DOI:** 10.3390/ijms19061682

**Published:** 2018-06-06

**Authors:** Mara Fornasarig, Raffaella Magris, Valli De Re, Ettore Bidoli, Vincenzo Canzonieri, Stefania Maiero, Alessandra Viel, Renato Cannizzaro

**Affiliations:** 1 SOC di Gastroenterologia Oncologica, Centro di Riferimento Oncologico IRCSS, 33081 Aviano, Italy; raffaella.magris@cro.it (R.M.); smaiero@cro.it (S.M.); rcannizzaro@cro.it (R.C.); 2 SOSD Immunopatologia e biomarcatori Oncologico, Centro di Riferimento Oncologico IRCSS, 33081 Aviano, Italy; vdere@cro.it; 3 SOC di Epidemiologia, Centro di Riferimento Oncologico IRCSS, 33081 Aviano, Italy; bidolie@cro.it; 4 SOSD di Anatomia Patologica, Centro di Riferimento Oncologico IRCSS, 33081 Aviano, Italy; vcanzonieri@cro.it; 5 SOSD Oncogenetica e Oncogenomica Funzionale, Centro di Riferimento Oncologico IRCSS, 33081 Aviano, Italy; aviel@cro.it

**Keywords:** gastric cancer, lynch syndrome, familial adenomatous polyposis (FAP), helicobacter pylori infection, autoimmune gastritis, fundic gland polyps (FGPs)

## Abstract

Lynch syndrome (LS) and familial adenomatous polyposis (FAP) are autosomal dominant hereditary diseases caused by germline mutations leading to the development of colorectal cancer. Moreover, these mutations result in the development of a spectrum of different tumors, including gastric cancers (GCs). Since the clinical characteristics of GCs associated with LS and FAP are not well known, we investigated clinical and molecular features of GCs occurring in patients with LS and FAP attending our Institution. The Hereditary Tumor Registry was established in 1994 at the Department of Oncologic Gastroenterology, CRO Aviano National Cancer Institute, Italy. It includes 139 patients with LS and 86 patients with FAP. Patients were recruited locally for prospective surveillance. Out of 139 LS patients, 4 developed GC—3 in the presence of helicobacter pylori infection and 1 on the background of autoimmune diseases. All GCs displayed a high microsatellite instability (MSI-H) and loss of related mismatch repair (MMR) protein. One of the FAP patients developed a flat adenoma, displaying low-grade dysplasia at the gastric body, and another poorly differentiated adenocarcinoma with signet ring cells like Krukenberg without HP infection. LS carriers displayed a risk of GC. The recognition of HP infection and autoimmune diseases would indicate those at higher risk for an endoscopic surveillance. Regarding FAP, the data suggested the need of suitable endoscopic surveillance in long survivals with diffuse fundic gland polyps.

## 1. Introduction

Lynch syndrome (LS) and familial adenomatous polyposis (FAP) are the most frequent syndromes in hereditary colorectal cancer (CRC), causing, respectively, 6% and 1% of all CRC. Extracolonic neoplasms are often observed in these syndromes and upper gastrointestinal (GI) malignancies are an important cause of death.

LS is an autosomal dominant disorder caused by germline mutations in one of the mismatch repair (MMR) genes (MSH2, MLH1, MSH6, PMS2) or the EpCAM gene that mainly determines CRC risk. However, these mutations evolve into a spectrum of different extracolonic tumors. Endometrial cancer is the most frequent extracolonic cancer followed by urothelial, small bowel, and gastric cancers (GCs) [1,2]. MSH2, MLH1, and EpCAM carriers display a 46% risk of developing CRC and a 57% risk of endometrial cancer by the age of 75 years. Much lower is the risk for GCs (13%), but it is still higher compared to the 1% risk in the general population. MSH6 carriers instead have a lower risk for CRC (15%), endometrial cancer (46%), and also for GCs (<3%). Tumor tissue of CRC and extracolonic cancers in an LS setting show two peculiar molecular features: microsatellite instability (MSI) that is characterized by length alteration within simple, repeated DNA sequences called microsatellites, and loss of MMR protein expression at immunohistochemical analyses. These two molecular features are useful screening markers to identify patients with LS [3]. Guidelines are mainly focused on CRC prevention, suggesting colonoscopy starting at the age of 20–22 years with a two-year interval for recognition and removal of the precancerous lesions, i.e., adenomatous polyps. There is no consensus for extracolonic cancer prevention and for GCs surveillance [3,4,5]. GCs in this setting are usually of intestinal type, showing high microsatellite instability (MSI-H) and a loss of relative MMR protein expression. The natural history and pathological transformation pathway are unknown. However, helicobacter pylori (HP) infection represents a predisposing clinical condition to GCs, and its eradication is recommended. Gastric adenomas are rarely observed in LS; however, a recent study reported PGA in 3 patients out of 15 cases of LS GC [6].

FAP is an autosomal dominant hereditary disease caused by germline mutations in the adenomatous polyposis coli (APC) gene with 80–100% penetrance, leading to the development of hundreds to thousands colorectal adenomatous polyps starting in teenage years and CRC at an average age of 39 years. The correlation between genotype and phenotype has been highlighted. The APC gene encodes 2844 aminoacids in 15 exons. The mutation cluster region, located between 1250–1464 codon, is associated with florid and more aggressive colonic polyposis, whereas mutations in 5′ and 3′ regions are related to an attenuated FAP (AFAP) with a lower number of colonic adenomas. Mutations between 140–1309 are linked to papillary thyroid carcinoma and between 1399–1580 to desmoids tumors [7,8]. CRC has been considered an inevitable consequence in the natural history of FAP. Guidelines suggest colonic surveillance starting before teenage and prophylactic colectomy is the recommended treatment for preventing cancer when adenomatous polyposis is not endoscopically manageable or in the presence of adenomas with high-grade dysplasia [5]. Beside CRC, duodenal adenomas and carcinomas and desmoids tumors are the most frequent cause of morbidity and mortality in FAP. Presently, upper gastrointestinal (GI) endoscopy has been included in the surveillance starting at 25 years of age for staging duodenal polyposis, and the intervals are based on the Spigelman score determined by the number and grade of dysplasia of duodenal adenomas. However, GCs have also been reported [9]. Differences in frequency were seen between Western and Eastern countries [10]. A recent paper has described GCs in 0.5% of FAP patients from a US registry [11], while among Japanese FAP patients, the prevalence of GC has been reported to be from 2.8% to 15.5% [12]. The types of gastric lesions associated with GCs in FAP are: fundic gland polyps (FGPs), gastric foveolar-type gastric adenoma, gastric adenoma, and pyloric gland adenoma (PGA) [13]. FGPs are present in more than 60% of FAP patients and they usually display biallelic inactivation of the APC gene often with foci of dysplasia or microadenomatous polyps of the foveolar epithelium [14]. However, malignant progression in FGPs is uncommon and the lifetime risk of GC is reported to be in the range of 0.5–1%. Intestinal-type adenomas are rare (1–2% of gastric polyps) in Western countries compared to Asia (40–50% of gastric polyps) [15]. A strong correlation between gastric adenomas and atrophic gastritis secondary to HP infection has been showed in Japanese FAP patients [12,16]. PGAs have been recently recognized as a new polyp subtype, developing on the mucosa with pyloric metaplasia [17]. They have been found in 6% of FAP patients and high grade dysplasia is seen in 10% of cases. Out of the FAP setting, PGAs have been found in association with autoimmune metaplastic atrophic gastritis [18].

Since the clinical characteristics of GCs associated with LS and FAP are still not well understood, in our study we investigated clinical and molecular features of GCs occurring in these patients attending our Institution.

## 2. Results

**Figure 1:**
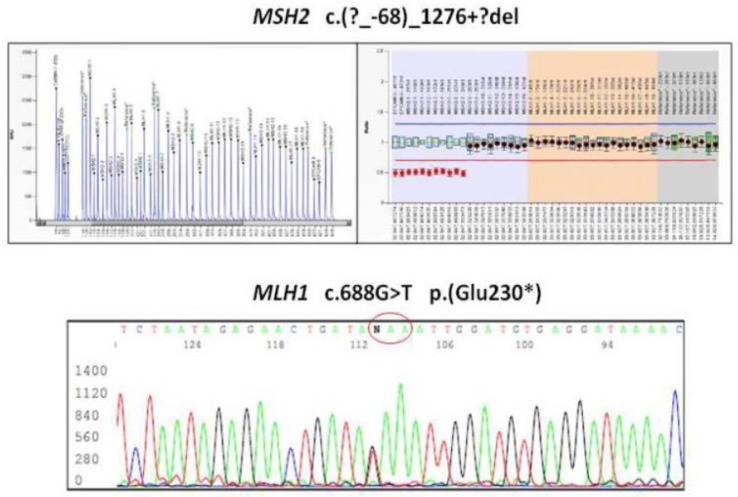
Representative mismatch repair gene mutations. **Top** panels: MLPA electropherogram (**left**) and analysis of MLPA data with the Software Coffalyser v.140721.1958 (MRC-Holland, Amsterdam, Holland). (NET (**right**) showing the MSH2 c.(?_-68)_1276+?del variant, corresponding to a large deletion encompassing exons 1–7. **Bottom** panel: Sanger sequencing showing the MLH1 c.688G>T variant producing the truncated p.(G1u230*) protein. The mutated codon (GAA > TAA) is circled in red.

**Figure 2:**
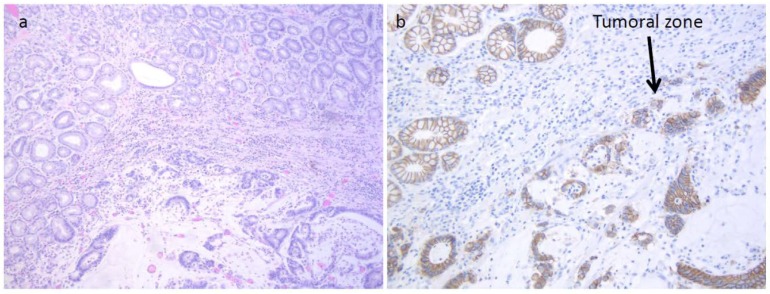
Patient 1: (**a**) Neoplastic cells showing diffuse solid growth and focal vague glandular appearances. H&E staining, original magnification 10×; (**b**) Reduction of E-cadherin expression by immunohistochemical staining, original magnification 20×.

**Figure 3:**
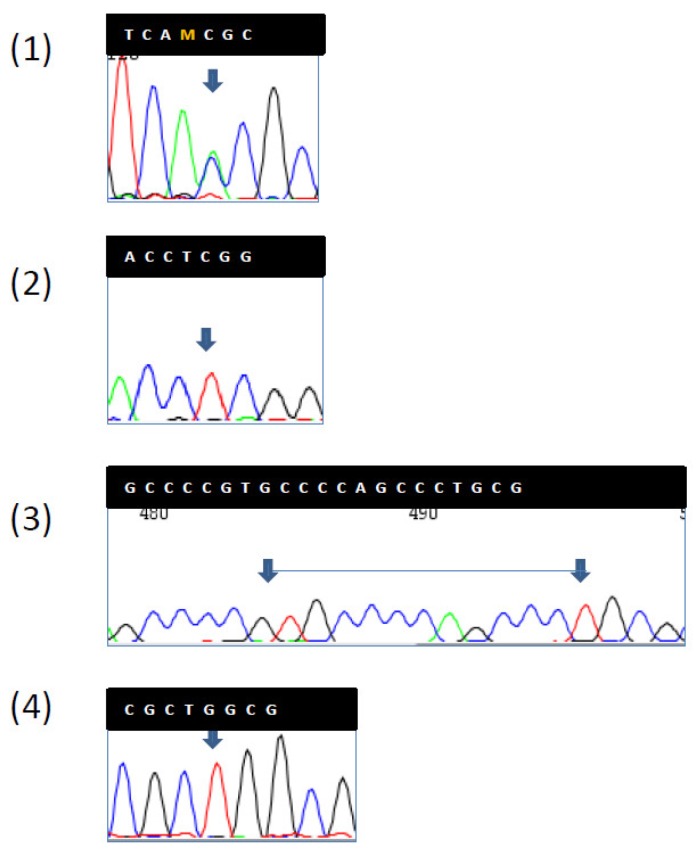
Four germline mutations, one of uncertain significance and three presumably benign were found in the *CDH1* gene. Sequence chromatograms. (**1**) mutation of uncertain significance located in the promoter region of the E-cadherin CDH1 gene (rs16260 C/A); (**2**) mutation located in the intron 1 (rs3743674 T/T); (**3**) insertion located in the intron 1 (rs74406246 ins); (**4**) mutation located in the codon 13 (rs2076 T/T).

In LS, CRC was the most frequently diagnosed primary cancer (82 patients, 59%), followed by endometrial cancer (55% out of 83 women). Out of 82 patients with CRC, 24 had multiple synchronous lesions with a total of 131 CRCs diagnosed. Mucinous histotype was present in 55.7% (72) of CRCs, and the remaining ones were well or poorly differentiated adenocarcinomas. GC was the fourth most frequently diagnosed extracolonic cancer. Out of 139 patients, 18 (13%) showed HP infection and 4 patients (2.9%) (2 male, 2 female) developed a GC. All GCs displayed MSI-H and the loss of related MMR protein. The four patients with GCs are described as follows. Patient 1 was a 53-year-old man carrying the MLH1 mutation (Table 1, Figure 1) who developed an HP infection negative diffuse-type adenocarcinoma (T2N0) at the fundus (Figure 2a). The tumor displayed the reduction of cytoplasmic expression of E-cadherin (Figure 2b). Four years before diagnosis, he had two PGAs removed at the body with high-grade dysplasia. On that setting, an autoimmune gastritis was diagnosed with already atrophic gastritis and a deficit of acid secretion. Since the reduction of E-cadherin expression in tumor tissue and mutations in the CDH1 gene (codifying for E-cadherin) are strongly associated with diffuse-type adenocarcinoma, we also performed the sequence analysis of germline CDH1 gene. The patient showed common polymorphisms and nonpathogenetic variants (Figure 3). This patient has not developed CRC yet, but he underwent removal of four adenomas with low-grade dysplasia during his colonoscopic surveillance.

**Figure 4:**
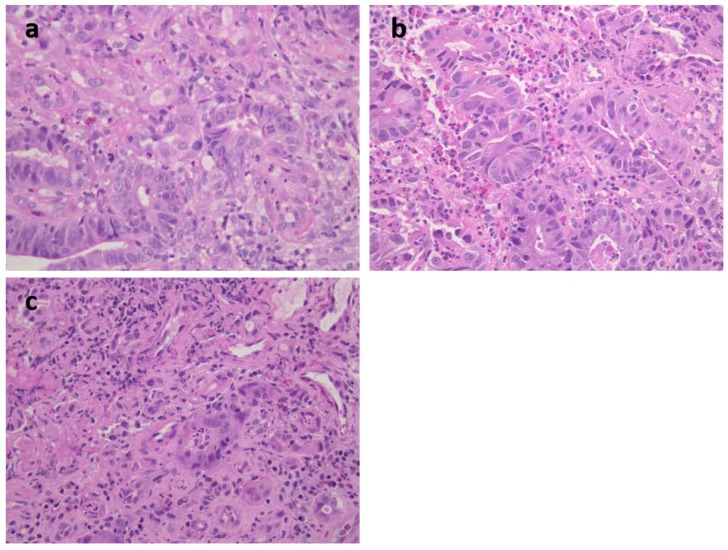
(**a**) Patient 2: moderately differentiated intestinal-type adenocarcinoma; (**b**) Patient 3: moderately differentiated intestinal-type adenocarcinoma; (**c**) Patient 4: moderately and poorly differentiated intestinal-type adenocarcinoma; H&H staining, original magnification 400×.

Patient 2 (Table 1, Figure 1) was a 73-year-old female carrying an MSH2 mutation with adenocarcinoma moderately differentiated intestinal type at antrum (T2N1) (Figure 4a). She had previous diagnoses of multiple primary cancers (CRC at ascending colon at age 27 years, endometrial cancer at 45 years, CRC at descending colon at 56 years, and urothelial cancer at 65 years). Patient 3 was a 62-year-old male carrying an MSH2 mutation with an adenocarcinoma poorly differentiated intestinal type (T2N0) at corpus (Table 1 and Figure 4b) associated with HP infection. He had already developed multiple CRCs at the ascending colon at age 34 years and at the sigmoid colon at 53 years. Patient 4 was a 40-year-old female carrying an MSH2 mutation with adenocarcinoma intestinal type poorly differentiated at corpus (T2 N1) and HP infection (Table 1 and Figure 4c). A previous CRC at the ascending colon was diagnosed at age 32 years.

**Figure 5:**
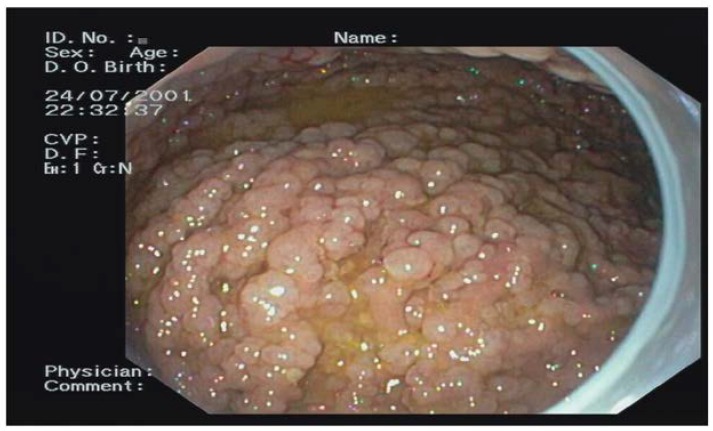
Diffuse fundic gland polyps.

**Figure 6:**
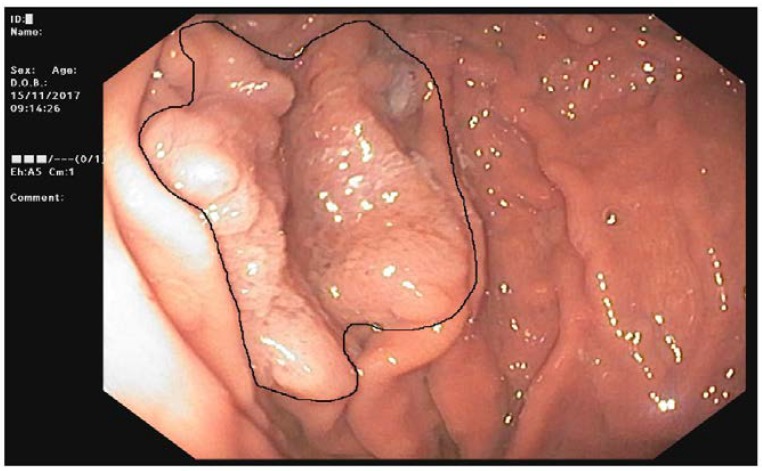
Flat adenoma in fundic gland polyposis.

Out of the 74 FAP patients under surveillance, 64 (94%) were diagnosed with FGPs and 11 (16%) with a diffuse carpeting at fundus and body (Figure 5). After 15 years of prophylactic colectomy, a 54-year-old male developed a flat adenoma displaying low-grade dysplasia at the gastric body about 25 mm in diameter in the presence of diffuse FGPs (Table 1, Figure 6). An adenocarcinoma poorly differentiated with signet ring cells like Krukenberg (T3N+) without HP infection was diagnosed in a 72-year-old female with a previous prophylactic colectomy at age 43 years. She had few FGPs without gastric adenomas and she developed ovary metastasis two years after gastrectomy (Table 1).

## 3. Discussion

This study reports the clinical and molecular features of a small cohort of patients with FAP, LS, and GC who were recorded in the Hereditary Tumor Registry of the Department of Oncologic Gastroenterology at CRO Aviano. The small sample size necessarily implies a limitation of the effectiveness of our report. However, a substantial interesting point comes from our cases since all patients have been genetically characterized, come from the same geographical area, and the surveillance procedures have been performed at our Institution.

Regarding LS, MSI-H and the absence of MMR proteins corresponding to the germline mutation are the molecular features of GCs when they are related to this genetic disease [1,19]. GC has been reported to be one of the most frequent extracolonic malignancies in MSH2 and MLH1 mutation carriers with an estimated lifetime risk up to 13% and a frequency ranging from 1.6% in a Dutch registry to 3.1% in Korea and 10.9% in Finland, with a mean age of 56 years in Western countries and 46 years in Eastern countries [1,2,6]. A correlation with familial GC history had been shown only in Korea. Our regional cancer registry, Friuli Venezia Giulia Cancer Registry, has reported a cumulative lifetime risk of GC of 2.6% for males and 1.2% for women in the general population, which is lower than that estimated for LS patients. The frequency of GC in our cohort was 2.9% in the range of previous reports.

GCs displayed features of LS, such as MSI-H and the lack of MMR protein expression. Our cases had mutations in MLH1 and MSH2, the two genes mainly involved in GC risk [1,20]. The mean age of patients with GC was 57 years, and they did not have a GC family history. Three cases were of the intestinal type and one of the diffuse type. Intestinal GCs are often preceded by chronic atrophic gastritis with intestinal metaplasia and are related to environmental exposures such as diet, smoking, alcohol, and HP infection. In our series, HP infection was observed in all intestinal-type GCs and the diagnosis of cancer was made by upper GI endoscopy in a work up for symptoms. HP infection has been reported in about 20% of GCs in LS [2,6,21]. No differences in the frequency of HP infection were observed between LS carriers and the general population [21]. Diffuse-type GC was already reported in LS, with the highest frequency of 17% [2]. Our case of diffuse-type GC was diagnosed during a follow-up for atrophic gastritis associated with diffuse intestinal metaplasia caused by autoimmune diseases. The patient was positive at antiparietal cell antibodies. Atrophic gastritis was histologically ascertained as was an acid-secretion deficit by pepsinogen I and a low dose of vitamin B12. Two PGAs with high-grade dysplasia were endoscopically removed at the body and diffuse-type cancer was diagnosed four years later. PGAs are a distinct entity, they can derive from deep gastric mucous glands and they are likely accompanied by a background of intestinal metaplasia and autoimmune gastritis. They can evolve into invasive adenocarcinoma displaying pyloric gland differentiation [22]. PGAs were described in 3 out of 15 cases of GCs by Lee [6]. Two cases were diagnosed at the edge of the tumor, and in one case, PGA was removed two years before the GC diagnosis. However, we do not have any information on the association with autoimmune diseases. In autoimmune gastritis inflammatory aggression affects oxytocin glands at the gastric body and fundus leading to atrophia, and parietal cells may be replaced by cells containing mucus, similar to the intestinal ones, i.e., intestinal metaplasia. Atrophic gastritis with intestinal metaplasia is a recognized precancerous condition, usually predisposed to intestinal type adenocarcinoma or to carcinoid tumors in the setting of autoimmune gastritis [23]. GC was diagnosed, although there was a close surveillance of yearly endoscopy since our patient had already developed precancerous lesions as PGAs and he was an LS carrier. Thus, we hypothesized a role of CDH1 in the pathogenesis of GC because the diffuse type is uncommon in autoimmune gastritis; although CDH1 is involved in hereditary diffuse GC, pathogenetic mutations were not found in the molecular analysis. GCs were mainly diagnosed after previous CRCs, and one patient only developed GC as a primary cancer, but we must take into account that he was under colonic surveillance and multiple adenomas were removed. GC guidelines suggest surveillance in countries at higher risk of GC, such as Asian countries, or treatment of HP infection [3,4,5]. Our GC developed in the two clinical conditions leading to atrophic gastritis: autoimmune gastritis and HP infection. Our results confirmed current guidelines to search for and treat HP infection. Regarding the role of autoimmune gastritis in LS patients, our findings must be confirmed by further research, as it was identified only in one patient in a small cohort.

FAP is a genetic disease with an approximately 100% risk of CRC. Since mortality of CRC has considerably shrunk after the introduction of genetic testing and prophylactic colectomy, surveillance for extracolonic cancer has been suggested [5]. In our cases, metastatic CRCs were diagnosed in 12 patients because they were not aware of their genetic disease; no CRC was diagnosed in the other patients. They started surveillance at the appropriate age and they were treated with prophylactic colectomy or they were still under colonoscopic surveillance. Upper GI endoscopy is recommended for staging duodenal polyposis and the intervals are based on the Spigelman score determined by the number and grade of dysplasia of duodenal adenomas, regardless of gastric polyposis. However, recent data has emerged on GCs developed on the background of FGPs [11]. Unlike Far East populations, GC was uncommon in FAP [10] and our data are in line with previous reports. Actually, our case of GC was not related to FAP; it was a Krukenberg tumor, which is a rare metastatic signet ring cell tumor in the ovary. The stomach is the primary site in the majority of Krukenberg tumor cases, followed by carcinomas of the colon, appendix, and breast. The tumor must display mucin-secreting signet ring cell carcinoma in the dense fibroblastic stroma of the ovary to be defined as a Krukenberg tumor. Instead, another patient developed a large gastric adenoma that was located at the gastric body among diffuse FGPs. This adenoma probably could represent the precancerous lesions on a dysplasia foci or small adenoma on FGP background as reported in [11]. Our cases displayed APC mutations in the same region (between 685–2040 codon) as described by Walton [11]; this finding is, however, controversial because the same correlation was not found with adenomas. Comparing others features reported in GC cases, our patients did not have desmoid tumors or diffuse duodenal adenomas. GC diagnoses in the Japanese FAP cohort differed from British ones as well as our case for multicentric adenocarcinoma foci associated with HP infection and early onset. So far, guidelines on FAP have not accomplished gastric surveillance in patients with diffuse FGPs. Different intervals and new tools such as high-definition and narrow-binding light endoscopes must be considered for detection of small adenomas among diffuse hyperplastic polyposis.

In conclusion, our results confirm the risk of GC in LS carriers and indicate that surveillance programs should include investigation of HP infection. In addition, further studies are still needed the shed light on the role of autoimmune diseases. Regarding FAP, our limited data did not allow us to draw definitive conclusions, but previous reports suggest a suitable endoscopic surveillance for long survivals with diffuse gastric FGPs in Western countries.

## 4. Materials and Methods

### 4.1. Patients

One hundred thirty-nine patients with LS and 86 patients with FAP were recorded in the Hereditary Tumor Registry, that was established and approved on the 21th September 1994 by the Institutional Board of CRO-IRCCS, National Cancer Institute of Aviano (PN), Italy. All registered patients or their legal guardian, provided informed written consent. Ethical guidelines for research involving human subjects were respected. 

#### 4.1.1. LS Patients

**Figure 7:**
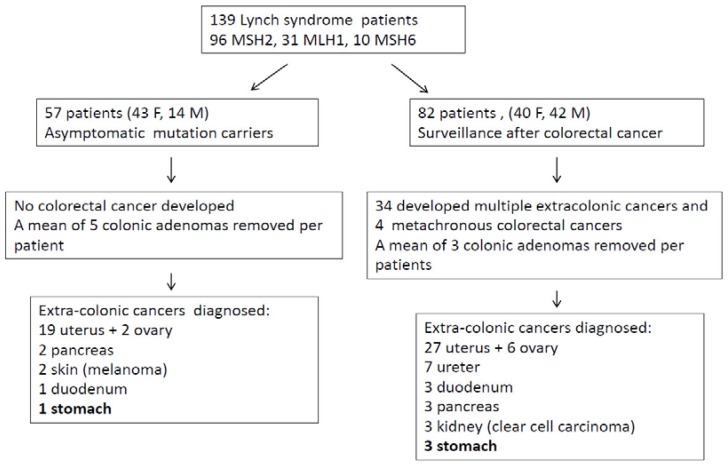
Lynch syndrome patients and cancer registered.

Out of 139 LS patients (83 females, 56 males; mean age 53 years), 33 had mutation in MLH1, 10 in MSH6, and 96 in MSH2. Fifty-seven patients entered surveillance because of asymptomatic mutation carriers and 82 patients were in follow-up after a previous CRC (Figure 7). The average follow-up time was 10.5 years (2–26 years). The surveillance program consisted of colonoscopy starting at age 20 years, repeated every 2 years until the age of 40 years, and annually thereafter. Gynecological surveillance for women started at age 30 years with abdominal ultrasound, with urinary cytology beginning at age 35 years and repeated every 2 years. Upper GI endoscopy was performed according to guidelines only in patients with a family history of GC or because of symptoms until 2005, then the procedure was extended to all carriers staring at age 35, repeated every 3 years. Nowadays, European and American guidelines still give different suggestions [3,4,5]. Figure 7 describes patient number and types of cancers recorded during the follow-up.

#### 4.1.2. FAP or AFAP Patients

**Figure 8:**
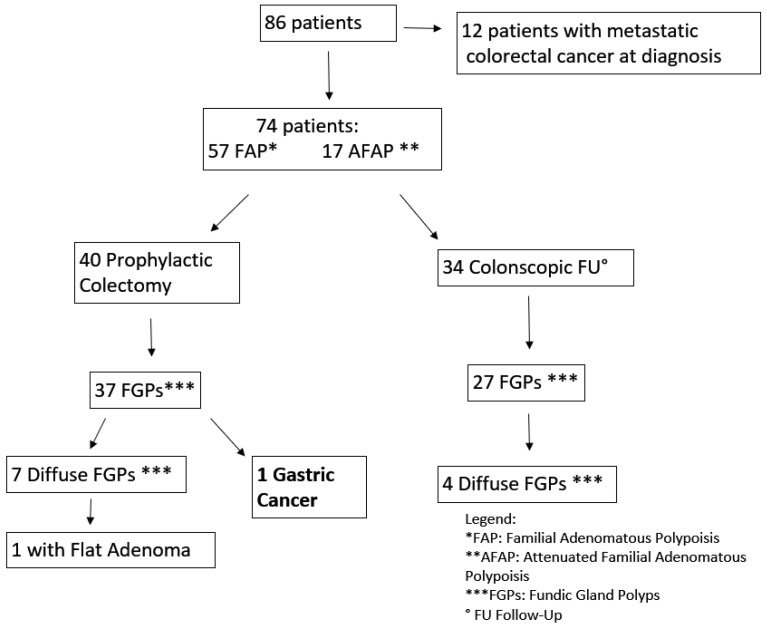
Familial adenomatous polyposis (FAP) patients.

FAP patients (42 female, 44 male; mean age 46.7 years) had mutations identified in the APC gene. Sixty-two patients (32 female, 36 male; mean age 45.6) had mutations between 1250–1464 codon related to more aggressive polyposis and 18 in regions associated with the attenuated form (AFAP) (Figure 8). Upper GI endoscopy was performed according to guidelines starting at age 25 years or at the time of prophylactic colectomy. Twelve patients had a metastatic CRC at FAP diagnosis. Seventy-four patients are still under surveillance (57 with classical FAP and 17 with AFAP) (Figure 2). The average follow-up time was 11.6 years (1–36 years). Forty patients were under surveillance after their prophylactic colectomy and 34 were still in colonoscopic follow-up. FGPs were diagnosed in 64 patients out of 74, with 11 showing a diffuse carpeting of polyps at the fundus and body and 63 displaying a small number of polyps. The endoscopic intervals were determined by the burden and grade of dysplasia of duodenal adenomas classified according to the Spigelman score, varying from every 4 years if no polyps were found to every 3–6 months for diffuse duodenal adenomas or adenomas with high grade dysplasia.

### 4.2. Histology and HP Infection Status

GCs were classified according to Laurén into intestinal, diffuse, mixed, and indeterminate types, whereas WHO classification identifies five main types, i.e., tubular, papillary, mucinous, signet ring, and mixed carcinomas and a two- or three-tiered differentiation grading. Chronic gastritis, atrophy, intestinal metaplasia, and HP infection were evaluated and graded according to the Sydney classification. HP status was verified by conventional stainings (including H&E and special stains like Giemsa).

### 4.3. Mutational Analysis

Screening for constitutional point mutations of the three main DNA MMR genes (MSH2, MLH1, and MSH6) and APC gene was carried out on blood DNA by standard procedures of exon-by-exon amplification of the whole genomic region and flanking intron borders, followed by single-strand conformation polymorphism and/or direct bidirectional Sanger sequencing on an AB3130 xl Sequencer (Applied Biosystems/Thermofisher, Foster City, CA, USA) or by next generation sequencing targeting a custom gene panel on a MiSeq platform (Illumina, San Diego, CA, USA). Multiplex-Ligation Dependent Probe Amplification analyses (MLPA, MRC-Holland, Amsterdam, The Netherlands) were also used to detect large gene deletions/duplications.

Screening for mutations of the *CDH1* exons and neighboring intronic sequences was performed using polymerase chain reaction (PCR) with previously described primers and reaction conditions [20]. In short, 15 PCR reactions were performed for a full mutational screening of all 15 exons and splice junctions of the *CDH1* gene. Amplified PCR products were sequenced on the Applied Biosystems 3130 automated sequencer (Applied Biosystems, Foster City, CA, USA) using the Big Dye v3.1 Terminator Cycle Sequencing Kit (Life Technologies, Monza, Italy) and sequence data were aligned and analyzed using CodonCode Aligner software [24].

### 4.4. Microsatellite analysis

Genomic DNA was obtained from blood and from paraffin-embedded or frozen tissues. Standard MSI analysis was performed on paired tumor–normal tissue DNA samples using the original Bethesda panel of microsatellite markers (*BAT26*, *BAT25*, *D2S123*, *D5S346*, *D17S250*) or the mononucleotide panel (MSI Analysis System, Promega, WI, USA) including BAT26, BAT25, NR21, NR23, and MONO27. Fluorescent-labeled PCR products were separated by capillary electrophoresis using an ABI3130xl sequencer and evaluated with the GeneMapper software (version number 5, Applied Biosystems/Thermofisher, Foster City, CA). Criteria for definition of MSS and MSI were according to the Bethesda guidelines [25].

### 4.5. Immunohistochemistry (IHC)

MMR protein: formalin-fixed and paraffin-embedded sections from tumor blocks were stained by an automated method on the Ventana BenchMark Ultra. MLH-1 (M1), MSH2 (G219-1129), MSH6 (44) mouse monoclonal primary antibodies (Roche/Ventana), and PMS2 (EPR3947) rabbit monoclonal antibody (Roche/Ventana) were used to qualitatively identify human DNA MMR proteins. Lesions were considered positive for protein inactivation when a complete absence of nuclear staining was evident in tumor cells with concomitant nuclear staining of adjacent normal epithelial and stromal cells.

E-cadherin: the formalin-fixed, paraffin-embedded tumor block was cut into 4-μm-thick sections for H&E and immunostaining. Immunohistochemistry was performed by using the mouse monoclonal antibody against human E-cadherin (clone 36, Ventana Medical System, Tucson, Arizona). Lesions were considered positive for E-cadherin in the presence of membrane staining.

## Figures and Tables

**Table 1:** Molecular and pathological features of gastric cancer and adenoma in Lynch syndrome and FAP.

Mutated Gene	Mutation	Age at Onset	Gender	Histology	Stage
*MLH1*	c.688G>T p.(Glu230*)	53	M	Diffuse	T2N0
*MSH2*	c.(?_-68)_(*272_?)del p.(?)	73	F	Intestinal HP+	T2N1
*MSH2*	c.2334C>A p.(Cys778*)	62	M	Intestinal HP+	T2N0
*MSH2*	c.(?_-68)_1276+?del p.(?)	40	F	Intestinal HP+	T2N1
*APC*	c.1863-1866del p. (Thr621fs*8)	54	M	adenoma	
*APC*	c.1495C>T p.(Arg499*)	72	F	Krukenberg	T3N3

## Abbreviations

AFAP	Attenuated Familial Adenomatous Polyposis
APC	Adenomatous Polyposis Coli
CRC	colorectal Cancer
FAP	Familial Adenomatous Polyposis
FGP	Fundic Gland Polyps
GC	Gastric Cancer
HP	Helicobacter Pylori
IHC	Immunohistochemistry
LS	Lynch Syndrome
MMR	Mismatch repair
MSI-H	High Microsatellite instability
PGA	Pyloric Gland Adenoma
PCR	Polymerase Chain Reaction
